# Supplementary material for: Status and future prospects for mobile phone-enabled diagnostics in Tanzania
Source: PLOS Digit Health. 2024 Aug 9;3(8):e0000565. doi: 10.1371/journal.pdig.0000565 (PMC11315315; doi:10.1371/journal.pdig.0000565)
Supplement: S1 Questionnaire — Our key objectives were: I. Understanding Digital Health for Infectious Disease in Low Resource Settings; II. Understanding Data Integrity and Security in Digital Health in Tanzania; III. Exploring the Relationship between Data and Healthcare Policy; IV. Mobile phone Devices for Data Acquisition and Communication in Tanzania; and V. Capacity Strengthening—Educational Training underpinning Mobile Health. (DOCM) [file pdig.0000565.s001.docm]

**Mobile Phone enabled Diagnostics for infectious Disease Diagnosis for Low-Cost Tools for Digital Health in Tanzania.**

**Preamble**

This questionnaire is used in helping to understand the landscape of smartphone use in Tanzania and their potential application as m-health devices.

Our key objectives are:

1. Understanding Digital Health for Infectious Disease in Low Resource Settings
2. Understanding Data Integrity and Security in Digital Health in Tanzania;
3. Exploring the Relationship between Data and Healthcare Policy;
4. Mobile phone Devices for Data Acquisition and Communication in Tanzania;
5. Capacity Strengthening - Educational Training underpinning Mobile Health.

The questionnaire will take 5-10 minutes to answer and we shall not take personal details

**Questionnaire**

What is the name of the Health Facility/ Vet clinic?

……………………………………………………………………………………………………

Level........................................................................ District………………………….......

Village…………………………………………...

What is the catchment population?

……………………………………………………………………………………………………

Would you identify this facility as?

Urban

Rural

What is your profession?

Nurse

Doctor

Radiologist

Biomedical Engineer

ICT personnel

Veterinary Doctor

Veterinary technician

Other please specify …………………………………………………………………………...

What are your qualifications?

O-Level certificate

A-Level certificate

Diploma

Advanced Diploma

Degree

Masters

PHD

Age

Gender

What is your role at this facility?

Director

Medical superintendent

In-charge

Supervisor

Other specify…………………………………………………………………………

How many years have you used a computer?

| Never | 1year | 2years | 3years | 4years | 5years |
| --- | --- | --- | --- | --- | --- |

Do you have access to a smart phone?

What type of smart phone do you currently use?

iPhone

Techno

Samsung

Other please specify ………………………………………….

Please specify the model………………………………………….

What smartphone have you used before this current one?

iPhone

Techno

Samsung

Other please specify ………………………………………….

Please specify the model………………………………………….

What Apps do you use most for video calling?

WhatsApp

Imo

Messenger

Vyber

Other please specify …………………………………………………………...

What do you use most for messaging?

Text messages

Messenger

Instagram

WhatsApp

Other please specify………………………………………………………………….

What do you use most for notes and documenting?

Microsoft word

Google keep

Other please specify………………………………………………….

How often do you use your smartphone in a day?

| Less than 1hour | 1hour | 2hours | 3hours | 4hours | 5hours |
| --- | --- | --- | --- | --- | --- |

Have you ever used a web and mobile application for M-health and telemedicine? If yes, for what did you use it for?

……………………………………………………………………………………………………………………………………………………………………………………………………………………………………………………………………………………………………………………………………………………………………………………………………………………..

How widely are they used in your community and for what?

……………………………………………………………………………………………………………………………………………………………………………………………………………………………………………………………………………………………………………….

What are your thoughts/experience on web and mobile applications for M-health and Telemedicine?

……………………………………………………………………………………………………………………………………………………………………………………………………………………………………………………………………………………………………………………………………………………………………………………………………………………..

If we designed an M-health diagnostic tool, what would be your number one priority for it to have/do?

………………………………………………………………………………………………………………………………………………………………………………………………………………………………………………………………………………………………………………………
