# Supplementary material for: Status and future prospects for mobile phone-enabled diagnostics in Tanzania
Source: PLOS Digit Health. 2024 Aug 9;3(8):e0000565. doi: 10.1371/journal.pdig.0000565 (PMC11315315; doi:10.1371/journal.pdig.0000565)
Supplement: Attachment — Submitted filename: PLOS Reviewer Recommendation and Comments for Manuscript Number PDIG-D-23-00248.docx [file pdig.0000565.s002.docx]

Grammatical corrections:

Lines:

40 - that “the” majority

42 - them “to” apply

43 - health applications. (Add period at end of sentence)

59 - In this study, we aimed (Add comma after study)

62 - were “aware” of mobile phone diagnostics

64 - smartphone, however, have never used (Add comma after however)

71 - one, therefore, enabling (Add comma after therefore)

73 - equipment not equipment’s

74 - and, therefore, not (Add commas before and after therefore)

74 - majority of the population who live in remote areas (remove comma between population and who)

76 - community “settings”

87 - also “increases”

96 - malaria (9), where the Smartphone (space between malaria and (9); no space between (9) and comma)

97 - decision support, “and” data storage

132 - and “a” number of animal …The proportion of (not proportional)

134 - resulting into “a” study sample size of 288

154 - mobile health “enabled” “diagnostics” were done

165 - smartphone “enabled” devices

166 - in “the” future among

173 - clinical practice for “diagnosis” of disease

185 - for more than five “years”.

189 - source of “patient” education materials

199 - communicable diseases. (Add period at the end of sentence)

214 - who used “smartphones”

216 - Additionally, medical doctors (Add comma after “Additionally”)

219 - users of “smartphones”

221 - this has “a” consequential impact

222 - potentially impact the performance “of” (delete the word “on”)

223 - old fashion “smartphones” are likely

228 - Interestingly, the majority (Add comma after “Interestingly”)

231 - health care “professionals” own “smartphones”

233 - study done in “the” USA

264 - and ownership “of” “smartphones”

268 - on the use of “smartphones”

270 - implication os “the” use of “smartphones” for medical diagnosis

272 - deployment of “smartphone-based” medical diagnostics

Questions about the paper:

Lines:

104-105 - will the structured questionnaire or the questions asked within be provided with this paper? Was the qualitative information gathered from the questionnaires coded and categorized appropriately?

105 - why were two papers not written separately - one for medical staff and another for veterinary staff? Perhaps the paper would have been better focused had the authors or researchers concentrated their efforts in gathering information from one or the other group. Maybe an idea for a future paper.

113 - Were the answers from the focus group discussions coded and categorized appropriately? Was coding qualitative data used in your research and will the data be made available as part of the publication of this paper?

154-155 - Please provide samples of the semi-structured questionnaire with open-ended and closed questions.

164 - Please ensure that the data in the tables, figures, and frequencies are presented in the final draft of the manuscript prior to submission or publication.

166-168 - Was this narrative information considered unstructured qualitative data? And does “summarized thematically” mean that the information was coded? Please make sure to provide evidence of the link between the analysis of this information to the quantitative data.

Table 3: Human health professionals column - includes information that is not “priority diseases” but diagnostic labs/tests/tools - may want to re-label Table 3 to reflect the inclusion of both diseases and diagnostic tests (e.g. Microscope, Ultrasound, Pregnancy test). Correction from Diabetics to diabetes.

Table 3: Animal Health Professionals column - includes only priority diseases. Will the final draft have information on diagnostic labs/tests/tools used by veterinarians, as well?

210-212 - What is the significance of including this information in your discussion? How is it relevant to the study of the use of mHealth applications or smartphones as POC devices? Perhaps more elaboration of this information and its tie-in to mHealth is beneficial to readers.

246 - Are pregnancy tests and blood pressure considered priority diseases? Is pregnancy a disease? Or pathophysiologies associated with pregnancy (e.g. gestational diabetes, pregnancy-induced hypertension, pre-eclampsia/eclampsia, placental abruption, placenta previa, HELLP)? Mortality rate of high-risk pregnancies? Blood pressure as related to hypertension and cardiovascular diseases? Which ones?
