# Supplementary material for: Status and future prospects for mobile phone-enabled diagnostics in Tanzania
Source: PLOS Digit Health. 2024 Aug 9;3(8):e0000565. doi: 10.1371/journal.pdig.0000565 (PMC11315315; doi:10.1371/journal.pdig.0000565)
Supplement: Attachment — Submitted filename: Response to reviewers.docx [file pdig.0000565.s003.docx]

Dear Editors: PLOS Digital Health.

We would like to thank the reviewers for careful and thorough reading of this manuscript and for the thoughtful comments and constructive suggestions, which have helped to improve our manuscript.

In response, we have addressed all the comments and made updates to the work presented in this manuscript, including additional analysis to assist correlation of different variables and formatting references, as recommended by reviewers 1 & 2.

In the text below we provide a point-by-point response to the comments received from the reviewers and journal editor. We have made changes throughout the paper that are indicated in the tracked version of our revised paper. Formatting changes are not shown in track to improve readability.

Best wishes

Theonest Ndyetabura (on behalf of all authors)

Section Editor’s comments

**Journal Requirements:**

1. Please amend your detailed Financial Disclosure statement. This is published with the article. It must therefore be completed in full sentences and contain the exact wording you wish to be published.

a. State the initials, alongside each funding source, of each author to receive each grant.

b. State what role the funders took in the study. If the funders had no role in your study, please state: “The funders had no role in study design, data collection and analysis, decision to publish, or preparation of the manuscript.”

c. If any authors received a salary from any of your funders, please state which authors and which funders.

If you did not receive any funding for this study, please simply state: “The authors received no specific funding for this work.”

***Response:*** *Financial Disclosure statement has been amended, now reads as “This work was supported by funds from the Engineering and Physical Sciences Research Council (EPSRC), UKRI grant reference: EP/T029765/1, JC.* *The funder had no role in study design, data collection and analysis, decision to publish, or preparation of the manuscript.”*

2. Please provide separate figure files in .tif or .eps format only and remove any figures embedded in your manuscript file. Please also ensure that all files are under our size limit of 10MB.

For more information about figure files please see our guidelines:
<https://journals.plos.org/digitalhealth/s/figures>
<https://journals.plos.org/digitalhealth/s/figures#loc-file-requirements>

***Response:*** *Journal requirements for figures have been applied in figures embedded in this manuscript. Figure files are now in tif format and the size is less than 10MB. Map has been removed from the manuscript and is now provided in supplementary information (Figure 1).*

3. Some material included in your submission may be copyrighted. According to PLOS’s copyright policy, authors who use figures or other material (e.g., graphics, clipart, maps) from another author or copyright holder must demonstrate or obtain permission to publish this material under the Creative Commons Attribution 4.0 International (CC BY 4.0) License used by PLOS journals. Please closely review the details of PLOS’s copyright requirements here: PLOS Licenses and Copyright. If you need to request permissions from a copyright holder, you may use PLOS's Copyright Content Permission form.

Please respond directly to this email or email the journal office and provide any known details concerning your material's license terms and permissions required for reuse, even if you have not yet obtained copyright permissions or are unsure of your material's copyright compatibility.

Potential Copyright Issues:

Figure 1: please (a) provide a direct link to the base layer of the map (i.e., the country or region border shape) and ensure this is also included in the figure legend; and (b) provide a link to the terms of use / license information for the base layer image or shapefile. We cannot publish proprietary or copyrighted maps (e.g. Google Maps, Mapquest) and the terms of use for your map base layer must be compatible with our CC-BY 4.0 license.

Note: if you created the map in a software program like R or ArcGIS, please locate and indicate the source of the basemap shapefile onto which data has been plotted.

If your map was obtained from a copyrighted source please amend the figure so that the base map used is from an openly available source. Alternatively, please provide explicit written permission from the copyright holder granting you the right to publish the material under our CC-BY 4.0 license.

Please note that the following CC BY licenses are compatible with PLOS license: CC BY 4.0, CC BY 2.0 and CC BY 3.0, meanwhile such licenses as CC BY-ND 3.0 and others are not compatible due to additional restrictions.

If you are unsure whether you can use a map or not, please do reach out and we will be able to help you. The following websites are good examples of where you can source open access or public domain maps:
* U.S. Geological Survey (USGS) - All maps are in the public domain. ([http://www.usgs.gov](http://www.usgs.gov/))
* PlaniGlobe - All maps are published under a Creative Commons license so please cite “PlaniGlobe, [http://www.planiglobe.com](http://www.planiglobe.com/), CC BY 2.0” in the image credit after the caption. (<http://www.planiglobe.com/?lang=enl>)
* Natural Earth - All maps are public domain. (<http://www.naturalearthdata.com/about/terms-of-use/>) "

***Response****: We appreciate for opportunity to provide support regarding use of maps. We have provided description and a link in the map legend of how the map was created and accessed. We believe the information provided meets the PLOS Digital Health journal requirement. In case the provided information is not sufficient we will appreciate for your help in this.*

Additional Editor Comments (if provided):

The manuscript was interesting. Please address the following comments in your revision.

1-      Please follow the journal instructions for referencing style.

***Response****: We thank the editor for the comments, we have updated our references to meet the journal requirements.*

2-      Some terms such as smartphone are started with capital letters. It is better to revise the manuscript and be consistent in writing.

***Response****: We have carefully revised the manuscript to improve grammatical issues and inconsistencies.*

3-      The results section presents some simple descriptive statistics. I suggest the researchers to conduct more statistical analysis to find the correlation between different variables.

***Response****: We have performed further analysis to find out correlation between variables and presented our findings in table 3 which is included in revised manuscript.*

4-      In Table 1, please use “sex” instead of “Gender”.

***Response****: Changes have been made as suggested.*

5-      Please present the limitations of the study before the conclusion section.

***Response****: Changes have been made as suggested.*

**Reviewers' comments:**

**Reviewer #1:**

Status and future prospects for mobile phone-enabled diagnostics in Tanzania
This is an interesting study of smartphone use among veterinary and medical staff in Tanzania, with an emphasis on mHealth utilization.

The authors should consider paying for editing the paper for minor English grammar issues. Although the meaning is clear, there are some places where the grammar seems odd.

***Response:*** We thank the reviewer for their comments. English grammar issues have been reviewed in details by all authors.

Abstract: Use headers in the abstract (Introduction, Methods, Results, Discussion, Conclusion).

***Response****: Abstract is in headers i.e., Introduction, Methods, Results, Discussion and Conclusion sections as suggested.*

Please spell out UTI.

***Response****: UTI is now spelled out, reads as “urinary tract infections”*

Data collection: Who were the interviewers? It's common to include the initials of the co-authors who did the interviews.

***Response****: A description of who conducted interviews and initials of their names have been added in the methodology section, now reads as* *“An assessment of awareness, literacy, opinion about mobile phone enabled diagnostics among medical and veterinary workers was done using guided semi-structured questionnaires, in-depth interview and focused group discussion administered to the study participant by NT, KN, EK, AL, DK, IK, DM, and JV”.*

Were the responses input into the software by the study subjects or by the interviewers?

***Response***: *Responses input into the software (ODK) were done by interviewers at the point of interview. We have revised the data collection section in order to improve clarity and to incorporate questions raised by reviewers regarding data collection and entry process.*

Statistical analysis: Consider changing the first few words to read “Our analysis used a sequential explanatory design…”

***Response*:** *We thank the review for advice to change the first few words of statistical analysis section. We have made changes as suggested.*

Who did the thematic analysis of the qualitative interviews? Were the recommended themes reviewed by other authors? How were final decisions made in cases of disagreement about the themes?

***Response:*** *We have now provided information on approaches on thematic analysis.*

Table 1: Please spell out ICT.

***Response****: ICT in table 1 have been spelled out, now reads as “Information and Communication Technology”.*

Table 2: Consider putting the results under “Time of using smart phone per day” and “Duration of usage of mHealth applications” into numerical order. It’s a little confusing to have “over 5 hours” as the first response category and “5 hours” as the last response category. Similarly, it’s confusing to have “More than 5 Years” appear prior to “3 Years.”

***Response:*** *We thank the reviewer for the observation and suggestion on data organization issues in table 2. We have organized responses in numerical order as suggested.*

Results: Please further describe the thematic analysis of the qualitative interviews and focus groups. Currently, there is only a listing of priority diseases, which appears to be the response from an open-ended question.

***Response****: We thank the reviewer for this suggestion, we have provided further description in the methods section on thematic analysis of the qualitative interviews and focus group discussion. We have also provided addition analysis as indicated in revised table 3*.

Discussion: Consider making the discussion section a bit less of a repetition of the results. Start with a short paragraph summarizing the main results. The comparison with other studies is interesting but does not require the results to be re-described in much detail. For Jones et al. 2011, please use a numbered citation. Please write out what L MICs means.

***Responses:*** *Discussion section has been updated and all suggestions has been clearly considered.* *References have been formatted as per journal requirements.*

The “Limitation of the study” section should appear before the “Conclusion” section.

***Response****: We thank the reviewer for suggestion, the Limitation section have now been moved to appear before conclusion as suggested.*

Data availability statement: This journal may require a specific statement as to availability, specifically, placement of data in a shared repository. Please check the Journal requirements.

***Response:*** *Data availability statement has been revised and associated link has been added.*

**Reviewer #2:**

It would have been beneficial to the reviewer to have had the structured questionnaires available along with the manuscript upon review. Please ensure that they are included in the final submission. Ensure that all relevant data is included and made available for public use upon publication of the manuscript.

***Response***: *All data collection tools including structured questionnaires and link to the raw de-identified data has now been provided.*

Included with this review is an attachment in MS Word doc format with the reviewer's recommendation and comments. As far as the manuscript being presented in an intelligible fashion and written in standard English, I have cited lines from the manuscript where grammatical corrections may be made to improve the flow of ideas written on paper. These are mere recommendations which the authors may use as guidelines for revision.

***Response****: We have worked on grammatical and language issues to improve our manuscript.*

Please read the reviewer's questions about certain areas of the paper, more importantly where quantitative, unstructured data may have been collected and the nature of how that information was coded, categorized, and summarized. Please include your procedures of how this was done if applicable.

***Response****: We have added information on analysis of descriptive data and how coding was done.*

**Grammatical corrections:**

***Response****: We have worked on grammatical and language issues to improve our manuscript including those listed below. Line numbers may have shifted in both clean and marked manuscript versions.*

Lines:

40 - that “the” majority ------------------------------------------Inserted “the”

42 - them “to” apply--------------------	-------------------------Inserted “to”

43 - health applications. (Add period at end of sentence)…… Added period

59 - In this study, we aimed (Add comma after study)………Added comma

62 - were “aware” of mobile phone diagnostics………………deleted ness to retain aware

64 - smartphone, however, have never used (Add comma after however)…….added comma

71 - one, therefore, enabling (Add comma after therefore)….Added a comma

73 - equipment not equipment’s……………Deleted ‘s to retain equipment

74 - and, therefore, not (Add commas before and after therefore)…Added a comma before

74 - majority of the population who live in remote areas (remove comma between population and who) …. a comma removed

76 - community “settings” ……. Added an “S “

87 - also “increases”……………Added an “S”

96 - malaria (9), where the Smartphone (space between malaria and (9); no space between (9) and comma)…. Removed space

97 - decision support, “and” data storage

132 - and “a” number of animal …The proportion of (not proportional) : added an “a” and delete an “al”

134 - resulting into “a” study sample size of 288: Added an “a”

154 - mobile health “enabled” “diagnostics” were done: a “d” and “s” accordingly

165 - smartphone “enabled” devices: Replaced an “r” with a "d”

166 - in “the” future among: Inserted “the”

173 - clinical practice for “diagnosis” of disease: changed from “diagnostics” to “diagnosis”

185 - for more than five “years”.: Added an “s” to read as years.

189 - source of “patient” education materials: Deleted an “s” to read as patient

199 - communicable diseases. (Add period at the end of sentence): Added a period at the end of sentence

214 - who used “smartphones”: Added an “s” now reads as smartphones

216 - Additionally, medical doctors (Add comma after “Additionally”): Have added a comma

219 - users of “smartphones”: Have added an “s”

221 - this has “a” consequential impact: Added an “a”

222 - potentially impact the performance “of” (delete the word “on”): Deleted “on”

223 - old fashion “smartphones” are likely : Have added an “s”

228 - Interestingly, the majority (Add comma after “Interestingly”): Added a comma

231 - health care “professionals” own “smartphones”: Added “s”

233 - study done in “the” USA: Added “the”

264 - and ownership “of” “smartphones”: Added “of”

268 - on the use of “smartphones”: Added “s”

270 - implication of “the” use of “smartphones” for medical diagnosis: Added “the” and “s” accordingly

272 - deployment of “smartphone-based” medical diagnostics

**Questions about the paper:**

Lines:

104-105 - will the structured questionnaire or the questions asked within be provided with this paper?

***Response*:** Yes, all data collection tools and de-identified raw data will be made available for public use and for the reviewers of this manuscript. Data access statement has been revised, access link to the data associated with this manuscript is now provided.

Was the qualitative information gathered from the questionnaires coded and categorized appropriately?

***Response****: Yes, description on how qualitative data was processed have now been included in the methods section. Sections of results and discussion have also been revised to accommodate updates made in the methods section*.

105 - why were two papers not written separately - one for medical staff and another for veterinary staff? Perhaps the paper would have been better focused had the authors or researchers concentrated their efforts in gathering information from one or the other group. Maybe an idea for a future paper.

**Response:** *We thank the reviewer and we appreciate for the comments and suggestions given regarding writing two papers from the work for this manuscript. Our broader aim for this survey was to collect initial information which will serve as baseline for future researches and guidelines in development of low cost and accurate smartphone operated digital devices for diagnosis of diseases in resources limited settings. The work for this survey is now being used as baseline in the European Union funded project (*[*https://didida-health.eu/*](https://didida-health.eu/)*) , which is a buildup project to the work of this manuscript.*

113 - Were the answers from the focus group discussions coded and categorized appropriately? Was coding qualitative data used in your research and will the data be made available as part of the publication of this paper?

***Responses:*** *Yes, description on how qualitative data was processed have now been included in the methods section. Sections of results and discussion have also been revised to accommodate updates made in the methods section. Data for this work will be made available and an access link to data pertaining to this manuscript have now been provided, please see data availability statement which includes the link for data accession.*

154-155 - Please provide samples of the semi-structured questionnaire with open-ended and closed questions.

***Response:*** *A sample of the semi-structured questionnaire with open-ended and closed questions is provided with this revised manuscript.*

164 - Please ensure that the data in the tables, figures, and frequencies are presented in the final draft of the manuscript prior to submission or publication.

***Response.*** *We have added data as recommended.*

166-168 - Was this narrative information considered unstructured qualitative data? And does “summarized thematically” mean that the information was coded? Please make sure to provide evidence of the link between the analysis of this information to the quantitative data.

***Responses****: See responses above.*

Table 3: Human health professionals’ column - includes information that is not “priority diseases” but diagnostic labs/tests/tools - may want to re-label Table 3 to reflect the inclusion of both diseases and diagnostic tests (e.g. Microscope, Ultrasound, Pregnancy test). Correction from Diabetics to diabetes.

***Response****: We have revised and updated tables, included phrases that medical condition other than diseases is covered.*

Table 3: Animal Health Professionals column - includes only priority diseases. Will the final draft have information on diagnostic labs/tests/tools used by veterinarians, as well?

***Response****: Table three has been changed, after further analysis, results regarding priority diseases now is presented as figures 2 and 3.*

210-212 - What is the significance of including this information in your discussion? How is it relevant to the study of the use of mHealth applications or smartphones as POC devices? Perhaps more elaboration of this information and its tie-in to mHealth is beneficial to readers.

***Response****: Discussion section has been revised to retain information that are significant our study.*

246 - Are pregnancy tests and blood pressure considered priority diseases? Is pregnancy a disease? Or pathophysiologies associated with pregnancy (e.g. gestational diabetes, pregnancy-induced hypertension, pre-eclampsia/eclampsia, placental abruption, placenta previa, HELLP)? Mortality rate of high-risk pregnancies? Blood pressure as related to hypertension and cardiovascular diseases? Which ones?

***Response****: We have carefully revised responses on priority diseases, now states as priority diseases or pathophysiology. We have performed further analysis on priority diseases, results are presented in figure 2 and 3.*
