# Supplementary material for: Status and future prospects for mobile phone-enabled diagnostics in Tanzania
Source: PLOS Digit Health. 2024 Aug 9;3(8):e0000565. doi: 10.1371/journal.pdig.0000565 (PMC11315315; doi:10.1371/journal.pdig.0000565)
Supplement: Attachment — Submitted filename: Response to reviewers_23 April 2024.docx [file pdig.0000565.s004.docx]

Dear Editors: PLOS Digital Health.

We would like to thank the reviewers for careful and thorough reading of this manuscript and for the thoughtful comments and constructive suggestions, which have helped to improve our manuscript.

In response, we have addressed all the comments by reviewers 3 & 5 and made updates to the work presented in this manuscript, including formatting tables, improving typographical and grammatical errors, matched Funding Information and Financial Disclosure Statement. We have further elaborated points which were just mentioned or briefly discussed in our previous manuscript version.

In the text below we provide a point-by-point response to the comments received from the reviewers. We have made changes throughout the paper that are indicated in the tracked version of our revised paper. Formatting changes are not shown in track to improve readability.

Thank you for your consideration

Theonest Ndyetabura (on behalf of all authors)

Response to the comments reviewers:

Reviewer #3:

**Comment:** The introduction outlines the need for information on smartphone use in disease diagnosis among healthcare professionals in Tanzania, but the objectives and hypotheses are not explicitly stated. Clearly defining these aspects.

***Response:*** *In response to your suggestion, we have revised the introduction to explicitly state the objectives and hypotheses of our study. The added sentence reads “To gather baseline information, we conducted a survey study aimed at assessing the current utilization of mobile phone-enabled diagnostics among healthcare professionals in Tanzania, identifying the challenges and barriers faced in implementing such diagnostics in the healthcare system, and exploring potential future prospects for integrating them into routine healthcare practices. Although our study was more exploratory in nature, we anticipated varying levels of awareness and adoption among healthcare professionals in different regions of Tanzania. We also expected barriers such as infrastructure limitations and technological literacy to hinder widespread implementation, but we see the potential for targeted interventions and capacity building efforts to overcome these barriers*.”

**Comment:** The study's sample size determination lacks detailed justification, and the distribution across regions appears arbitrary. The uneven distribution may affect the study's external validity and generalizability.

**Response:** *We acknowledge the importance of providing a detailed justification for the sample size determination and distribution across regions in our study. We have revised the section by providing detailed information and added reference to justify our careful considerations in determining samples size and distribution across regions. The revised section now reads as “The determination of the sample size was conducted through a comprehensive assessment of the estimated total of healthcare workers (66,348) and animal healthcare workers (8,943) in Tanzania, resulting in a combined population of 75,291 individuals [19] . This calculation took into account the proportion of smartphone users within the study population, which was estimated at approximately 75%, supported by existing literature and data on smartphone penetration in Tanzania. To ensure the external validity and generalizability of the findings, the sample size was further adjusted for each sampling region, resulting in a total of 305 instead of 288 individuals. The allocation of the overall sample size across regions was not arbitrary. Instead, it was meticulously designed to ensure adequate representation of the diverse geographical areas within Tanzania. Each region received a proportionate share of the sample size, allowing for the capture of variations in healthcare infrastructure, access to technology, and other contextual factors that could influence the adoption and implementation of mobile phone-enabled diagnostics.”* We hope are responses meets your expectations.

**Comment:** The study predominantly focuses on urban areas, potentially leading to sampling bias. Including rural areas is crucial for a comprehensive understanding of smartphone utilization in different settings.

***Response:*** *We acknowledge reviewer’s concern regarding the potential sampling bias introduced by predominantly focusing on urban areas in our research. While we fully recognize the importance of including rural areas for a comprehensive understanding of smartphone utilization. During our study design, after careful consideration we noted that current smartphone usage in these areas remains relatively low. We recognize the significance of tracking this trend as smartphone adoption steadily increases in rural communities, therefore we have clearly stated urban-centric focus as one of the limitations (under study limitation section) of our study and provided suggestions on the importance of extending research efforts to rural areas in the future study.*

**Comment:** The absence of a control group limits the ability to compare smartphone use among healthcare professionals with the general population, hindering a broader perspective.

***Response:*** *We appreciate the reviewer's insightful comment regarding the absence of a control group in our study. It is indeed a valid point that comparing smartphone use among healthcare professionals with the general population could provide a broader perspective on mobile phone-enabled diagnostics in Tanzania. Our study focused specifically on human and animal healthcare professionals and their utilization of mobile phone-enabled diagnostics in Tanzania, considering that this particular group will likely be the first to adopt mobile phone-enable diagnostic technology before the general population. The primary aim was to assess the current status and future prospects within this specific group, rather than comparing them to the general population. In future research endeavors, incorporating a control group from the general population could offer valuable insights into how smartphone use for diagnostics differs between healthcare professionals and non-healthcare individuals. This comparative analysis could enhance the understanding of mobile health technology adoption and usage patterns across different segments of society. The absence of a control group is well stated as one of the limitations to our study.*

**Comment:**  **Add a few quotes or excerpts from the responses of healthcare professionals in the results and discussion** *sections.*

***Response****: We appreciate your suggestion to include quotes or excerpts from the responses of healthcare professionals in the results and discussion sections to provide further depth and insight into our findings. Below, we have incorporated examples of such quotes or excerpts as recorded from study participants.*

**Comment:** Make visual representations, such as word clouds, to present the thematic analysis findings in a more accessible format.

***Response****: We appreciate your suggestion to include visual representations, such as word clouds, to present our thematic analysis findings in a more accessible format. While we acknowledge the value of visual representations in enhancing the accessibility of research findings, we believe that our manuscript, in its current format, effectively communicates the results of our thematic analysis. Through clear and concise language, we have articulated the key themes and findings derived from our study, providing readers with a comprehensive understanding of the topic at hand. We have ensured that our revised manuscript is structured in a logical and organized manner, with clear headings and subheadings guiding the reader through the various sections. Additionally, we have incorporated relevant tables and figures to support the presentation of our data and analysis. We are confident that the revisions made to our manuscript have strengthened its clarity, coherence, and accessibility, and we hope that you will find the current presentation format satisfactory*.

**Comment:** The study briefly touches upon potential barriers such as awareness, policy guidelines, mobile data connectivity, and cultural practices influencing smartphone use for healthcare. However, a more in-depth exploration of these barriers, through qualitative interviews or additional survey questions, is needed.

***Response****: Thank you for this valuable comment. In our revised manuscript under different sections, we have explored and discussed barriers for implementation of mobile phone-enabled diagnostics in details.*

**Comment:** While the results are presented, there is a lack of critical discussion regarding the implications of the findings. For instance, the low utilization of smartphones for healthcare practices raises questions about the underlying reasons, and potential strategies to address these issues should be discussed.

**Response:** *We appreciate the reviewer’s insightful feedback regarding the need for a more comprehensive discussion on the implications of our research findings. In response to this valuable suggestion, we have expanded our manuscript to include a detailed analysis of why there is low utilization of smartphones for healthcare practices in Tanzania. We have identified several key factors contributing to this phenomenon, such as limited access to smartphones among healthcare providers, inadequate digital literacy skills, infrastructure challenges, and potential cultural barriers. Furthermore, we have proposed various strategies to address these issues, including targeted training programs to enhance digital skills among healthcare workers, initiatives to improve smartphone affordability and accessibility, collaborations with mobile network operators to expand network coverage in remote areas, and community engagement efforts to promote acceptance and adoption of mobile phone-enabled diagnostics.*

**Comment:** In Figure 1, please include a link to the terms of use or license information. This information is crucial for meeting the CC-BY 4.0 license requirements.

**Response:** *The link to the terms of use and license information has now been included.* *The map is under* [*Creative Commons Attribution 4.0*](https://creativecommons.org/licenses/by/4.0/)*, accessible* [*https://energydata.info/dataset/tanzania-region-district-boundary-2012*](https://energydata.info/dataset/tanzania-region-district-boundary-2012)

**Comment:** The methods section mentions thematic analysis, but details on the rigor, inter-rater reliability, or validation processes are lacking. A more robust description of the analytical approach would enhance the study's credibility. Also, you mentioned thematic analysis of open-ended responses, but the specific themes and subthemes identified from the responses are not explicitly presented. Providing a brief summary of the thematic analysis results could enhance clarity.

**Response:** *We appreciate the reviewers' comments regarding the need for a more detailed description of the analytical approach, particularly in relation to thematic analysis. We have enhanced the methods section in revised manuscript by providing a more robust explanation of the rigor, inter-rater reliability measures, and validation processes employed during the thematic analysis. We also included a brief summary of the thematic analysis results, outlining the key themes derived from the data to offer a clearer understanding of the findings.*

**Comment:** The study briefly mentions ethical approval and written consent but lacks discussion on potential ethical considerations associated with smartphone-enabled diagnostics, such as data security, patient privacy, and informed consent for diagnostic applications.

**Response:** *We appreciate your insightful feedback; in our revised manuscript we have incorporated a detailed analysis of the ethical considerations surrounding smartphone-enabled diagnostics in the discussion section.*

**Comment:** The study presents priority diseases for both human and animal healthcare professionals separately. A comparative analysis to identify commonalities and differences in priorities could provide valuable insights for developing integrated diagnostic solutions.

**Response***: Considering the limitations posed by the small number of animal healthcare professionals compared to human healthcare professionals in our study and in Tanzania at large, conducting a comparative analysis to identify commonalities and differences in priorities may present challenges and may not yield sufficiently robust insights for developing integrated diagnostic solutions at this time. The disparity in sample sizes between the two sectors could potentially skew the findings and compromise the validity of the comparative analysis. Therefore, while we acknowledge the potential value of such a comparative analysis in theory, we agree that given the current circumstances, it may not be feasible to pursue this approach in the context of our study. We appreciate the reviewer's consideration of these factors and will accordingly refrain from conducting a comparative analysis in the revised manuscript.*

**Comment**: Given the interdisciplinary nature of healthcare, emphasizing the potential benefits of multi-disciplinary collaboration in the discussion could highlight the holistic approach needed for successful implementation of mobile phone-enabled diagnostics.

**Response:** *Indeed, we acknowledge the interdisciplinary nature of healthcare and the importance of emphasizing the potential benefits of multi-disciplinary collaboration in our discussion. In response to your suggestion, we have considerably expanded the discussion section in response to several other comments. We believe the added information will provide readers of this manuscript/article with a more comprehensive understanding of the holistic approach required prior to deployment of mobile phone-enable diagnostic tools.*

**Comment:** While limitations are briefly mentioned, a more detailed discussion of the study's limitations is needed. For example, discussing the potential biases introduced by the predominantly urban sample and the exclusion of the general population could be valuable.

**Response:** *We appreciate your suggestion regarding the need for a more detailed discussion of the study's limitations. In response to your comment, we have provided a more comprehensive discussion of the limitations in our revised manuscript.*

**Comment**:  Concluding the paper with a section on potential future research directions could provide insights for researchers and policymakers. For instance, discussing the need for studies focusing on rural populations, exploring ethical considerations, and addressing regulatory challenges would contribute to the discussion.

**Response:** *In our revised manuscription we have provided detailed explanations and discussions including the need for studies focusing on rural populations, exploring ethical considerations, and addressing regulatory challenges of so forth.*

**Comment:** A dedicated conclusion section summarizing the key findings, implications, and potential next steps will provide a more conclusive ending to the paper.

**Response:** *We have revised and enriched our conclusion section with key information as advised, care has been taken not to repeat key findings/information already presented in the abstract section.*

**Reviewer #5:**

**Comment:** The concept of the study seems interesting as laying groundwork for next stages of digital engagement for Tanzania. There are still several typographical errors and grammatical inconsistencies, but the revision has addressed many of them. Conceptually there also seems to be some lack of cohesion with the introduction through to the conclusions which could use refinement.

**Response:** *We thank the reviewer for additional comments, we have addressed few addressed typographical errors and grammatical inconsistencies noted. We have addressed cohesion issues by improving introduction section and rewriting conclusion section.*

**Comment:** The author summary should not be a copy and pasted abstract starting in the second sentence and ending with the second to last, but rather how this work fits into the broader context of similar literature. The words "considerable disparity" carries connotations related to unfair treatment, would reconsider its use.
**Response:** *We thank the review for comments regarding authors summary. We have rewritten authors summary; we believe that our revised authors summary is detailed and well written.*

**Comment:** Introduction: would benefit from some substantiation of the value of mobile diagnostics. There is a sentence describing some examples, but with very few details. More information about the effectiveness of these tools in other areas or uptake of use would be helpful to understand the study's context.

***Response:*** *Thank you for providing valuable feedback on our manuscript. We appreciate your insightful suggestion regarding the need to substantiate the value of mobile diagnostics by providing more comprehensive examples and details about their effectiveness in other contexts. We have enhanced the introduction section as well as other sections of this manuscript, providing detailed information that we believe have greatly improved our manuscript.*

**Comment:** Methods: There were three modalities of data collection (questionnaires, in-depth interview, and focus groups), but how did these integrate with each other? What was sent to the Clinical Research Institute, a transcribed interview with the questionnaires or a post-processed form of theme assignment by the investigators? Table 2 seems like it is reporting the questionnaire entirely, what additional information did the interviews provide?
***Response:*** *We thank the reviewer for valuable comment. We have revised our manuscript add a few quotes/excerpts from the responses collected form in-depth interview and focus group discussion, these are found in the results and discussion sections.*

**Comment**: Methods, study area: what does "professionals based on literacy level providing constructive opinion on the use of mobile phone for health purposes" indicate? Did the veterinary clinics not have this?
***Response:*** *Professionals based on literacy level indicate level of current understanding and future prospects of use of mobile phone enabled diagnostics. In our survey we noted that animal healthcare professionals were less aware about mobile phone-enabled diagnostics compared to human health care professionals. In our revised manuscript we have address this issue in different sections.*

**Comments:** Methods, study subjects: Regarding the statement "based on their professional skills and their role at the health facilities," (lines 160-161) does this imply the selection wasn't based on professional licensing status?
***Response:*** *Yes, selection was not based on professional licensing status due to the fact that in Tanzania not all healthcare practitioner are licensed. Our study included licensed and non-licensed healthcare practitioners provided in their day-to-day activities are engaged in providing health services.*

**Comment**: Methods: The power calculation was not clear to me.
***Response:*** *We have revised this section; we believe our revised paragraph on power calculation will now be clear to you.*

**Comment**: Methods, data collection: the author initials and names listed in the manuscript may be more appropriate in the supplemental materials, but this may be a style difference.
***Response:*** *We thank you for the advice, we added this information in the text in response to another reviewer’s comment.*

**Comments**: Methods; there should be explicit statement of the primary and (if applicable) secondary outcomes. These seem to be mentioned tangentially in the data collection section.
***Response****: In response to other comments, we believe we have improved our manuscript, making methods clear and bold.*

**Comment**: Methods, statistical analysis: I would change the language of the analysis to be directly related to your outcomes. "To investigate the changes and trends on awareness and literacy and use of smartphone" does not provide enough information--is this referring to the logistic regression and awareness of mHealth specifically?
**Response:** *We thank you for advice. We have changed the language, now reads as “Our analysis used a sequential explanatory design whereby the quantitative data were descriptively analysed using python 3.6.0 [22] to ascertain on awareness and literacy of mobile phone-enabled diagnostics among human and animal healthcare professionals”*

**Comment:** There is not a mention of mHealth prior to writing about the logistic regression. mHealth is bigger than simple medical diagnostic tools, so this should be differentiated if awareness of this tool is to be a dedicated outcome.
**Response:** *Thank for valuable observation. We have added our primary outcome of interested in the sentences preceding logistic regression, we believe this has improved clarity of our sentence. The sentence now reads as “To establish correlation between different variables to our primary variable of interest; awareness of mobile phone-enabled diagnostics, we performed binary logistic and multinomial logistic regression ….”*

**Comment**: The statement about results in the statistical analysis section could be removed. The statistical analysis section has some sentences that seem out of order for the logical sequence.
***Response****: We have removed the statement about results in our statistics analysis section.*

Results:
The sentence "workers responded to a survey which aimed to assess their perception towards the use of smartphone in clinical practice for diagnosis of diseases/conditions" belongs in the methods section.
***Response:*** *We thank you for your comment. We have removed the mentioned sentence from the results section. We have not included in the method section because an equivalent sentence has already been added.*

**Comment**: Table 1 formatting should be consistent.
***Response****: Table 1 format has been improved and is now consistent.*

**Comment:** Table 2 formatting should be consistent: font and indentations are variable.
***Response****: Table 2 format has been improved and is now consistent.*

**Comment:** There is mention of the various uses of smartphones in healthcare practice, including accessing guidelines and patient education materials. If it was studied and is being reported, the distribution should be quantified. It may be helpful to include in Table 2, if so.
***Response:*** *We acknowledge the importance of quantifying this information on uses of smartphones in healthcare practice, we opted to provide detailed clarification and discussion throughout various sections of the manuscript rather than presenting them in Table 2. We believe that by integrating this information into the narrative, we can offer a more comprehensive understanding of the role of smartphones in healthcare practice to the readers of this manuscript.*

**Comment:** Changing the term "priority diseases" to "priority diseases/pathophysiology" still implies that pregnancy itself is a problem or harmful. If the domain is not referring to "complications of pregnancy," then the label should be changed.
***Response****: It is indeed the domain is not referring to complications of pregnancy. We have changed the term priority diseases/pathophysiology to “prevalent health condition”.*

**Comment**: In lines 267-272, this is an important foundation for why the specific phone apps were reported at all, but it needs more development for me to agree with the relevance.
***Response:*** *Thank for pinpointing our important foundation of our study. We have provided in our revised manuscript detailed explanations for why understand the most used apps is important to consider when developing diseases diagnostic apps. We hope you will find it more convincing.*

**Comments**: Some grammatical and typographical points to address (not comprehensive):

**Response:**

- The word "diagnostic" is an adjective and "diagnostics" is a noun. The phrase should either be "Point of care diagnostic technologies" or "Point of care diagnostics." ***We have made changes appropriately as suggested.***

- "focussed group" should be "focus group" ***changed from focused group to focus group.***
- In lines 34-35 of the abstract it is not immediately clear that the numbers following the region names are the distribution of participants. ***We have now clearly indicated the number following the region name that it represents the participants per region***.
- some semicolons are not used correctly: **This have been generally improved in our revised manuscript.**
- line 38, the number 97.4% should be in parentheses: ***97.4% is now in parentheses as recommended****.*
- The abstract discussion is a very long run-on sentence. ***We have revised and split the long sentence*.**
- The abstract conclusion is also a very long run-on sentence. ***We have revised and split the long sentence.***
- lines 83-84 the word "diseases" should be singular, or as applicable if the word "pathophysiology" was meant to be included: ***Changed from diseases to disease as recommended*.**
- line 96, "easy" should be "ease": ***Changed from easy to ease as recommended***
- line 116, "indepth" should be hyphenated: ***The word indepth has been hyphenated, now reads as in-depth***
- "m-health" is typically abbreviated "mHealth," but is also not consistent throughout the manuscript: ***m-health has been changed to mHealth throughout the manuscript.***
- line 133, the word "brackets" should be "parentheses": ***changed from brackets to parentheses as recommended***
- line 151 the word "proportional" should be "proportion": ***changed from proportional to proportion as recommended***
- line 212, the word "animals" should be singular*:* ***changed from animals to animal as recommended***
