# Supplementary material for: Status and future prospects for mobile phone-enabled diagnostics in Tanzania
Source: PLOS Digit Health. 2024 Aug 9;3(8):e0000565. doi: 10.1371/journal.pdig.0000565 (PMC11315315; doi:10.1371/journal.pdig.0000565)
Supplement: Attachment — Submitted filename: PDIG-D-23-00248R2_Response to the reviewer&Editor.docx [file pdig.0000565.s005.docx]

Dear Editors: PLOS Digital Health.

We would like to thank the reviewers for careful and thorough reading of this manuscript and for the thoughtful comments and constructive suggestions, which have helped to improve our manuscript.

In response, we have addressed few additional comments by reviewer 5 and made minor updates to the work presented in this manuscript, including formatting tables, improving typographical and grammatical errors. We have slightly updated funding information (in bold) in the manuscript and further matched funding information and financial disclosure statement.

The funding information now reads “*This work was supported by funds from the Engineering and Physical Sciences Research Council (EPSRC), UKRI grant reference: EP/T029765/1, JC.* ***This work is also part of a project that has received funding from the European Union's Horizon 2020 research and innovation programme under grant agreement No. 101057251 (DIDIDA), as well as from United Kingdom Research and Innovation Innovate UK (10052860).*** *The funder had no role in study design, data collection and analysis, decision to publish, or preparation of the manuscript*.”

We have done through review of all references and made updates on only two references, i.e. **13** and **37**. No article cited in this manuscript has been retracted.

In the text below we provide a point-by-point response to the comments received from the fifth reviewer. We have made minor changes that are indicated in the tracked version of our revised manuscript. Formatting changes are not shown in track to improve readability.

Thank you for your consideration

Theonest Ndyetabura (on behalf of all authors)

**Response to journal section editor and reviewer’s comments.**

**Section Editor:** Journal Requirements:

**Comment**: Please review your reference list to ensure that it is complete and correct. If you have cited papers that have been retracted, please include the rationale for doing so in the manuscript text, or remove these references and replace them with relevant current references. Any changes to the reference list should be mentioned in the rebuttal letter that accompanies your revised manuscript. If you need to cite a retracted article, indicate the article's retracted status in the References list and also include a citation and full reference for the retraction notice.

***Response****: Refence list has been reviewed. Only references 13 and 37 has been updated, no retracted articles have been cited in this manuscript.*

**Response to Reviewer #5:**

The substance of most comments has been addressed. Thank you for your attention to these details. There are still some areas requiring attention and typographical errors remaining.

**Introduction section:**
**Comment**: 107 - POC should have a following noun like "POC test" or "POC enzyme linked immunosorbent assay" without the "for"

***Response****: We have removed “for”*

**Commet**: 140 - correct the double space

***Response****: Double space corrected*

**Methods section:**

**Comment**: - Thank you for stating the variable of interest more directly in lines 232-233, but the sentence is still not clear due to the incorrect semicolon use. It should read something more like "The primary outcome was whether or not a person was familiar with mobile phone-enabled diagnostics." Why was both a binary and multinomial logistic regression model created? Either the primary outcome was binary or it was multi-class, but it can't be both. If two types of models were created for two different outcomes, then the second outcome also needs to be stated directly.

***Response****: We have split the sentence into two, improved clarity and corrected semicolon.*

**Comment**: - 232-235 should be broken up into more than one sentence

***Response:*** *The sentence has been broken into two as per recommended*

**Comment**: - The term mHealth should be defined before line 154 if it's going to be used interchangeably with POC diagnostics, probably in the Introduction.

***Response:*** *The term mHealth is defines in line 56 which is under keywords.*

-**Comments**: 201 Letters should not be capitalized

***Response****: now in small letter*

**Comment**: - 213 "to ascertain on awareness" is not the correct usage and has two spaces to correct
***Response****: The two spaces has been removed, to ascertain on awareness has been changed to “to assess awareness”*

**Results section**:
**Comment**: Table 1 still has inconsistent formatting. The category labels do not need to be in the middle of the table.

***Response:*** *Table 1 has been formatted and all category labels justified left*.

**Comment**: Table 3 title: would state the outcome in the title, the words "correlation" and "different parameters" are redundant, "mhealth" is inconsistent with prior usage

***Response****: Redundant words removed in the title of the table 3. Consistence ensured for mHealth usage*.

**Comment**: Table 3 is not referenced in the text; the results are also not discussed
***Response****: Table 3 is referenced and discusses, see lines 373-380.*

**Discussion section.**
**Comment**: The implications of the findings for the logistic regression model(s) should be included.

***Response****: Implication of the findings for the logistic regression models are included and well discussed. See lines 373-386.*

**Comments**: 342, 357, 402 the "-" is not needed

***Response****: “the” has been removed in suggested line numbers*

**Comment**: 361 "mobile health (mHealth)" should be defined earlier

***Response:*** *The term mhealth is defined in line 56, which is under keywords.*

**Comment**: 362 "LMICs" has already been defined as an abbreviation

***Response****: We have removed the definition for LMIC.*

**Comments:** 363 inconsistent formatting with the word "mhealth"

***Response:*** *consistence formatting has been done for the word mhealth.*

**Comments:** 367-368 "mobile health (mHealth)" has already been defined

***Response****: We have now retained the defined term*.

**Comment**: Tables and Figures don't need to be referenced with parentheses in the discussion section, just the results section

***Response****: We have removed parentheses for tables and figures in the discussion section.*

**Comment**: 415 "condition" should be plural

***Response****: Made plural*

**Comment**: 451 "Limitation" should be plural, "study" should be capitalized

***Response****: Made plural*
